# Clinical characteristics and laboratory biomarkers changes in COVID-19 patients requiring or not intensive or sub-intensive care: a comparative study

**DOI:** 10.1186/s12879-020-05647-7

**Published:** 2020-12-09

**Authors:** Anna Maria Cattelan, Eugenia Di Meco, Marco Trevenzoli, Alessia Frater, Anna Ferrari, Marco Villano, Federica Gomiero, Giovanni Carretta, Lolita Sasset

**Affiliations:** 1 Infectious Diseases Unit, Azienda Ospedale Università di Padova, Via Nicolò Giustiniani 2, 35128 Padova, Italy; 2 Information Technology System Unit, Azienda Ospedale Università di Padova, Padova, Italy; 3 Department of Directional Hospital Management, Azienda Ospedale Università di Padova, Padova, Italy

**Keywords:** COVID-19, SARS-CoV-2, Novel coronavirus, Disease severity, Outcome, Intensive care, Sub-intensive care

## Abstract

**Background:**

Identifying risk factors for severe novel-coronavirus disease (COVID-19) is useful to ascertain which patients may benefit from advanced supportive care. The study offers a description of COVID-19 patients, admitted to a general ward for a non-critical clinical picture, with the aim to analyse the differences between those transferred to the intensive (ICU) and/or sub-intensive care (SICU) units and those who were not.

**Methods:**

This observational retrospective study includes all COVID-19 patients admitted to the Infectious Diseases Unit. Clinical, laboratory, radiological and treatment data were collected. The primary outcome was a composite of need of transfer to the ICU and/or SICU during the hospitalization. Patients who did not require to be transferred are defined as Group 1; patients who were transferred to the ICU and/or SICU are defined as Group 2. Demographic, clinical characteristics and laboratory findings at the 1st, 3rd and last measurements were compared between the two groups.

**Results:**

303 were included. The median age was 62 years. 69 patients (22.8%) met the primary outcome and were defined as Group 2. The overall fatality rate was 6.8%. Group 2 patients were predominantly male (76.8% vs. 55.1%, *p* < 0.01), had a higher fatality rate (14.5% vs. 3.8%, *p* < 0,01), had more hypertension (72.4% vs. 44%, *p* < 0,01) and diabetes (31.9% vs. 21%, *p* = 0.04) and were more likely to present dry cough (49.3% vs. 25.2%, *p* < 0.01). Overall, chest X-ray at admission showed findings suggestive of pneumonia in 63.2%, and Group 2 were more likely to develop pathological findings during the hospitalization (72.7% vs. 17.2%, *p* = 0.01). At admission, Group 2 presented significantly higher neutrophil count, aspartate-transaminase and C-Reactive-Protein. At the 3rd measurement, Group 2 presented persistently higher neutrophil count, hepatic inflammation markers and C-Reactive-Protein. Group 1 presented a shorter duration from admission to negativization of follow-up swabs (20 vs. 35 days, *p* < 0.01).

**Conclusions:**

The presence of comorbidities and the persistent observation of abnormal laboratory findings should be regarded as predisposing factors for clinical worsening.

**Supplementary Information:**

The online version contains supplementary material available at 10.1186/s12879-020-05647-7.

## Background

The outbreak of severe acute respiratory syndrome coronavirus-2 (SARS-CoV-2) causing the human disease named coronavirus disease (COVID-19) was first reported in China in December 2019 and declared a pandemic by the World Health Organization in March 2020 [1]. The first known cases of local transmission were detected in Italy at the end of February 2020. The northern regions of the country initially have been the most affected by the outbreak [2]. To address the emergency, strict social containment measures have been adopted and health care systems have been reorganized to cope with the enormous increase in the numbers of acutely ill patients [3, 4].

Hospital admission rates for patients with COVID-19 may vary substantially between countries, because of the different prevalence in infection and community testing rates and non-homogeneous admission criteria. However, it is estimated that 10 to 20% of adults presents clinical conditions requiring hospitalization, and in the majority of the cases this is due to respiratory distress [5]. The decision about location of care and clinical management depends on various factors, including clinical presentation, disease severity, need for supportive care, presence of risk factors for severe Diseases, and conditions at home. While mild to moderate disease in low-risk patients can be managed at home or in primary or secondary level healthcare facilities, severe and critical diseases need tertiary hospitals where high dependency/sub-intensive (SICU) or intensive care units (ICU) are available [6]. Because the provision of intensive care and the availability of resources is limited [7], the identification of risk factors for severe infection is crucial for clinicians to identify patients who may benefit from aggressive supportive care and for health and government officials to adequately address local outbreaks.

The percentage of patients requiring ICU has ranged from 5 to 37% [8–13]. The most common reason for intensive care unit admission is hypoxemic respiratory failure leading to mechanical ventilation (MV) or non-invasive ventilation (NIV) [14]. In a large cohort study performed on ICU admitted patients in northern Italy 88% of the patients required MV and 11% NIV [15]. Patients admitted to intensive care units are reported to be older and predominantly male, and to have more frequently existing comorbidities such as hypertension, heart failure, renal disease, and obesity. Lower lymphocyte count, elevated serum troponin, C-Reactive Protein, D-dimer, and white blood cell count are also more commonly seen in patients presenting with severe infections [10, 12, 13, 16, 17].

The aim of our study is to describe the clinical characteristics and the dynamic changes of laboratory parameters of the patients admitted to the Infectious Diseases Unit (IDU) of the University Hospital of Padua. In order to identify possible predictors of clinical worsening we compared the results between the group of patients who required being transferred to the ICU and/or SICU during the course of the hospitalization and the patients who did not.

## Methods

### Study design

This is a retrospective observational study of prospectively collected data of adult patients hospitalized at the IDU of the University Hospital of Padua, Veneto region, Italy. The IDU is one of the general wards where COVID-19 patients are admitted with a clinical picture not requiring intensive support. We included all adult patients (aged ≥18 years), admitted between February 22 and May 20, 2020, with laboratory-confirmed SARS-CoV-2 infection and hospitalized for ≥24 h. In order to include only the patients initially hospitalized with a non-critical clinical picture, in the analysis the patients who required being transferred to the ICU/SICU within 12 h from admission and those that were transferred to the IDU after a permanence in the ICU/SICU are excluded.

We defined the primary outcome as a composite endpoint of need of transfer to the ICU and/or the SICU during the hospitalization. The group of patients who did not require to be transferred are defined Group 1; the patients who were transferred to the ICU and/or SICU are defined Group 2. Criterion for the transfer to the SICU was the need for NIV (defined as assisted ventilation that delivers positive pressure throughout the respiratory cycle with additional phasic increases in airway pressure, without the presence of an endotracheal tube [18]). Criteria for the transfer to the ICU were the need for MV or NIV and/or the occurrence of shock or organ failure.

Local ethics committees were notified about the study protocol. The study was performed according to the ethical guidelines of the Declaration of Helsinki (7th revision).

### Data collected

Demographic, clinical, laboratory and treatment data were extracted from paper and electronic medical records using a standardised data collection form.

Laboratory confirmation of SARS-CoV-2 infection was obtained by the detection in respiratory specimens (throat-swab) by an in-house real-time RT-PCR method according to Lavezzo et al. [19]. Throat-swabs were performed by using flocked swabs in liquid-based collection and transport systems (eSwab®, Copan Italia Spa, Brescia, Italy). Follow up swabs were performed every 3 to 5 days. A patient was considered negative for SARS-CoV-2 when two throat-swabs, performed in consecutive days, resulted negative. In the case patients were still positive at discharge, the repetition of follow-up swabs was performed through the territorial health services from which data were collected.

Laboratory tests included: blood count, CD4 T lymphocyte count, activated partial thromboplastin time (APTT), prothrombin time (PT), alanine aminotransferase (ALT), aspartate aminotransferase (AST), gamma-glutamyl-transferase (GGT), alkaline phosphatase (ALP), total bilirubin, urea, creatinin, glomerular filtration rate calculation (GFR), C-Reactive Protein (CRP), procalcitonin and D-Dimer. The treating physician, according to patients’ clinical needs, decided the frequency of repetition of laboratory tests.

In order to describe the dynamic changes of laboratory tests and possible early patterns indicating a deterioration of clinical conditions, the 1st measurement available (at admission), the 3rd measurement since admission, and the last measurement (before discharge or death) were analysed. The choice to consider the 3rd measurement was dictated by two main factors: 1) the 3rd measurement was always performed between the 3rd and the 5th day of hospitalization; 2) the median time from hospital admission to ICU/SICU transfer was of 5 days.

Laboratory tests to exclude the presence of bacterial infections (blood and urine cultures, pneumococcal and legionella urinary antigen tests, Chlamydia pneumoniae and Mycoplasma pneumoniae serology) were also performed at admission and during the hospitalization in case of a clinical suspicion of superinfections.

Chest radiographs were done for all patients at admission and repeated according to clinical needs. They were considered positive in case of evidence of singular or multiple consolidations and/or interstitial opacities.

Anti SARS-CoV-2 drugs administered during the hospitalization for > 2 days were recorded. Criteria for the choice of the therapeutic regimens for COVID-19 followed national guidelines [20] and physicians’ judgements. As a general rule, patients with comorbidities, age > 70 years, respiratory symptoms and/or evidence of pneumonia were treated with chloroquine (CQ) (500 mg orally twice daily) or hydroxychloroquine (HCQ) (200 mg orally twice daily). CQ and HCQ could be associated with lopinavir/ritonavir (400 mg/100 mg twice daily) or azithromycin (500 mg orally daily); standard duration of therapies was 5–7 days. In addition, tocilizumab and remdesivir were also used. Intravenous high dose glucocorticoids were used only in severe or critical patients. All patients, if not contraindicated, received thromboprophylaxis with daily low-molecular weight heparin.

### Statistical analysis

Continuous and categorical variables were presented as median (IQR) and n (%), respectively. We used the R software (version 3.6.2) to perform Mann-Whitney U test, χ^2^ test, or Fisher’s exact test to compare differences between Group 1 and Group 2 patients where appropriate. A *p* -value less than 0.05 was considered statistically significant.

## Results

A total of 325 adult patients were hospitalised with a confirmed diagnosis of COVID-19 in the study period. Twenty-two patients were excluded because they met the exclusion criterion of being admitted to the ICU or SICU within 12 h since admission or before to the permanence in the IDU. Therefore, a total of 303 patients were included in the analysis.

The median age was 62 years (IQR 50–74) and 182 (60.1%) were men. The fatality rate was 6.8%. All deaths were due to respiratory failure. The overall median duration of hospitalization was 9 days (IQR 6–16). 69 patients (22.8%) met the composite outcome. Of those, 15 were transferred to the ICU, 36 to the SICU and 18 to both of the units. The median time from hospital admission to the transfer was 5 days (IQR 2–13.5).

Table 1 summarizes the main demographics characteristics of the sample and the differences between the two groups of patients: group 2 patients were predominantly male (76.8% vs. 55.1%, *p* < 0.01), had a significantly higher fatality rate (14.5% vs. 3.8%, *p* < 0.01) and a longer hospitalization (18 vs. 7 days, *p* < 0.01). The difference between the median age of the two groups did not reach statistical significance (*p* = 0.06).

**Table 1:** Demographic characteristics and outcomes of non-ICU/SICU (Group 1) and ICU/SICU (Group 2) patients

	Available	All patients n.303	Group 1 n.234 (77.2%)	Group 2 n.69 (22.8%)	***p***
**Sex male**	100%	182 (60.1%)	129 (55.1%)	53 (76.8%)	< 0.01
**Age, years**	100%	62 (50–74)	60 (47–72)	68 (56–77)	0.06
**Death**	100%	19 (6.8%)	9 (3.8%)	10 (14.5%)	< 0.01
**Length of hospitalization, days**	100%	9 (6–16)	7 (5–12)	18 (14–26)	< 0.01
**Patients discharged**	100%	284 (93.7%)	225 (96.1%)	59 (85.5%)	
• Discharged at home		185 (65.1%)	157 (69.8%)	28 (47.5%)	0.02
• Transferred to a nursing home, community health facility, hospice		7 (2.5%)	5 (2.2%)	2 (3.4%)	0.70
• Transferred to a secondary level hospital		32 (11.3%)	21 (9.3%)	11 (18.6%)	0.03
• Transferred to a rehabilitation facility		17 (5.9%)	8 (3.5%)	9 (15.2%)	0.80
• Data not available		43 (15.1%)	34 (15.1%)	9 (15.2%)	

Data presented as median (IQR) or as percentage. In the second column data availability is also shown

*ICU* Intensive Care Unit, *SICU* Sub-intensive Care Unit

From the 284 discharged patients, 185 (65.1%) went home, while 7 (2.5%), 32 (11.3%) and 17 (5.9%) were transferred to a nursing home/community healthcare facility, a secondary level hospital or to a rehabilitation institute respectively. Group 2 patients were less likely to be discharged at home (47.5 vs. 69.8%, *p* = 0.02) and were more often transferred to a secondary level hospital (18.6% vs. 9.3%, *p* = 0.03) or to a rehabilitation institute (15.2% vs. 3.5%, *p* = 0.80) compared to Group 1.

Table 2 shows the clinical presentation at admission and the coexisting medical conditions. Fever (80.2%), dyspnoea (31.7%) and dry cough (30.7%) were the most common symptoms. Fever, which generally is considered being the most important symptom (as confirmed in this study), reached no statistically significant difference between the two groups (*p* = 0.73), while a significant difference was detected for the presence of dry cough (25.2% vs. 49.3%, *p* < 0.01).

**Table 2:** Clinical, radiological characteristics, treatments and follow-up swabs of Group 1 and Group 2 COVID-19 patients

	Available	All patients n. 303	Group 1 n. 234	Group 2 n. 69	***P***
**Co-existing medical conditions**	100%				
Hypertension		153 (50.5%)	103 (44.0%)	50 (72.4%)	< 0.01
Diabetes mellitus (pre-existing and newly diagnosed)		71 (23,.4%)	49 (21.0%)	22 (31.9%)	0.04
Chronic cardiac disease		44 (14,5%)	36 (15.4%)	8 (11.6%)	0.65
Chronic pulmonary disease		26 (8.6%)	19 (8.1%)	7 (11.1%)	0.67
Chronic gastrointestinal disease		26 (8.6%)	24 (10.3%)	2 (2.9%)	0.16
Active malignancy		26 (8.6%)	21 (9%)	5 (7.2%)	0.93
Transplant		3 (1.0%)	0 (0%)	3 (4.3%)	0.02
Urologic disorders		36 (11.9%)	28 (12%)	8 (11.6%)	0.90
Chronic kidney disease		15 (4.9%)	12 (5.1%)	3 (4.3%)	0.93
Obesity (BMI > 30)		51 (16.8%)	40 (17.1%)	11 (16.0%)	0.87
Overweight (BMI > 25)		154 (50.8%)	119 (50.8%)	35 (50.7%)	0.80
**Number of medical conditions**	100%				
0		101 (33.4%)	87 (37.2%)	14 (20.3%)	0.03
≥ 2		96 (31.7%)	71 (30.3%)	25 (36.2%)	0.47
**Symptoms at admission**	100%				
Fever		243 (80.2%)	187 (79.9%)	56 (83.2%)	0.73
Dry cough		93 (30.7%)	59 (25.2%)	34 (49.3%)	< 0.01
Productive cough		28 (9.2%)	24 (10.3%)	4 (5.8%)	0.54
Sore throat		16 (5.3%)	10 (4.3%)	6 (8.7%)	0.21
Dyspnoea		96 (31.7%)	69 (29.5%)	27 (39.1%)	0.20
Conjunctivitis		3 (1.0%)	0	3 (4.3%)	0.02
Diarrhoea		21 (6.9%)	17 (7.3%)	4 (5.8%)	0.81
Myalgia		23 (7.6%)	16 (6.8%)	7 (10.1%)	0.55
Arthralgia		13 (4.3%)	10 (4.3%)	3 (4.3%)	1
Malaise		41 (13.5%)	31 (13.2%)	10 (14.5%)	0.69
Dysgeusia		49 (16.2%)	40 (17.1%)	9 (13.0%)	0.52
Skin rash		13 (4.3%)	12 (5.1%)	1 (1.4%)	0.33
**Sat02 < 94% in air room at admission**	100%	134 (44.2%)	97 (41.4%)	37 (53.6%)	0.12
**Positive chest X-ray at admission**	92.3%	177 (63.2%)	140 (60.1%)	37 (78.7%)	0.15
**Positivization of chest X-ray during hospitalization**		24 (23.1%)	16 (17.2%)	8 (72.7%)	0.01
**Anti SARS-CoV-2 treatment**	100%				
Chloroquine or Hydroxychloroquine		183 (60.3%)	149 (63.7%)	34 (49.2%)	< 0.01
Lopinavir/ritonavir		88 (43.3%)	63 (26.9%)	25 (36.2%)	0.01
Remdesivir		19 (6.8%)	18 (7.7%)	1,0 (1.4%)	0.01
Tocilizumab		18 (5.9%)	12 (5.1%)	6 (8.7%)	0.15
Azithromycin		120 (39.6%)	99 (42.3%)	21 (30.4%)	0.01
**Antibiotic treatment iv**	100%	163 (53.8%)	111 (47.4%)	52 (75.3%)	< 0.01
**0xygen therapy**	100%				
Low or high flow systems^a^		168 (55.4%)	99 (42.3%)	69 (100%)	< 0.01
NIV		61 (20.1%)	0	61 (88.4%)	
MV		30 (9.9%)	0	30 (43.4%)	
**Negative follow up swab for SARS-CoV-2**^**b**^	93.7%	236 (83.1%)	182 (80.1%)	54 (91.5%)	0.70
**Days from hospitalization to negativization**	93.7%	22 (14–39)	20 (13.9–32)	35 (20–57)	< 0.01

Data presented as median (IQR) or as percentage. In the second column data availability is also shown

*ICU* Intensive Care Unit, *SICU* Sub-intensive Care Unit, *IQR* Interquartile Range, *BMI* Body Mass Index, *Sat02* Oxygen Saturation, *iv* intravenous, *NIV* Non-Invasive Ventilation, *MV* Mechanical Ventilation

^a^Nasal cannula, face-mask. ^b^Two negative throat-swabs obtained in consecutive days

Hypertension (72.4% vs. 44%, *p* < 0.01) and known or newly diagnosed diabetes (31.9% vs. 21%, *p* = 0.04) were more common among patients in Group 2 compared to Group 1. Patients with no comorbidities were less likely to belong to Group 2 (20.3% vs. 37.2%, *p* = 0.03).

Chest X-ray at admission to the IDU showed pulmonary lesions in 63.2% of the patients. No significant statistical differences were found between the two groups. Conversely, Group 2 patients were more likely to develop de novo pathological findings at the chest X-ray during the course of hospitalization (72.7% vs.17.2%, *p* = 0.01).

Laboratory findings at the 1st, 3rd and last measurements since admission are shown in Table 3 (see Additional file 1). Group 2 patients presented at admission a significantly higher neutrophil count, higher AST and CRP levels. At the 3rd measurement since admission, significant differences were found for white blood cell and neutrophil count, hepatic inflammation markers (AST, ALT, total bilirubin), and CRP. The GFR and the APTT resulted also significantly different between the two groups, but their median values were, in both cases, within the normal ranges. At discharge, Group 2 patients were found to have a significantly lower level of haemoglobin.

None of the patients resulted positive to the laboratory tests for bacterial infections (data not shown).

Table 2 shows data regarding antiviral drugs use. CQ or HCQ, lopinavir/ritonavir and azithromycin were the most common drugs, prescribed during the course of hospitalization to 60.3, 43.3 and 39.6% of the patients respectively. Group 2 patients were less likely to receive CQ or HCQ (49.2% vs. 63.7%, *p* < 0.01), azithromycin (30.4% vs. 42.3%, *p* = 0.01) and remdesivir (1.4% vs. 7.7%, *p* = 0.01), and more likely to receive lopinavir/ritonavir (36.2% vs. 26.9%, *p* = 0.01).

Among Group 2 patients, 88.4% required NIV and 43.4% MV.

As of June 1, 2020, 83.1% of patient resulted negative for SARS-CoV-2 at the follow-up swabs. The median duration from hospital admission to negativization (considering the date of the second swab) was 22 days. Patients of Group 1 presented a significant shorter duration to negativization compared to Group 2 (20 vs. 35 days, *p* < 0.01) (Table 1).

## Discussion

In this observational comparative study, clinical characteristics and laboratory biomarkers of a cohort of 303 patients with a confirmed SARS-CoV-2 infection and primarily hospitalized in the general ward of the Infectious Diseases Unit of Padua were analysed. 22.8% of the patients (Group 2) required to be transferred to the ICU and/or SICU and overall 19 patients (6.8%) died.

Our sample included only patients who presented at admission a non-critical clinical picture and did not primarily required intensive support. This could explain why our fatality rate was lower than those reported in recent European and American inpatient cohorts, ranging from 24 to 33% [11–13]. Moreover, population and patients’ characteristics and prevalence of community testing can also partly explain the differences. The admission rate to the intensive/sub-intensive care observed in our cohort is relatively high when compared with previous studies (rates comprised between 5 and 37%) [8–13]. It can be argued that a more extended aggressive support, especially in the form of a timely ventilator assistance, may guarantee a better management with higher chance of recovery of unstable or border-line patients.

Group 2 patients had a longer hospital stay and required more frequently to be transferred to a secondary level healthcare facility or to a rehabilitation institute compared to Group 1. These results may provide health officials and policymakers a better understanding on the interventions needed to properly address also long term consequences of local outbreaks.

Consistently with other studies [8–17], men were more represented than women and were at higher risk for severe disease. It has been reported that the reduced predisposition of females to viral infections could be attributed to the protection given by the X chromosome and sex hormones, which play an important role in the innate and adaptive immunity [21].

The median age was 62 years and the univariate analysis did not show a significant difference between the two groups of patients. This is possibly due to the narrow distribution of the patients in the age class 50–70 years (IQR = 47–74), which reflects the specific characteristics of our patients’ sample: older patients are more likely to present a critical clinical presentation and therefore to be directly admitted or precociously transferred to the ICU or SICU.

The presence of underlying medical disorders was associated with a greater risk to be transferred to the ICU or SICU. Hypertension and known or newly diagnosed diabetes were significantly more represented in Group 2. The three transplant patients of our cohort (a lung and two kidney transplants) required to be transferred to the SICU, but they all recovered. These data confirm that more fragile patients have a higher risk for severe disease and often require tertiary care management [22].

Fever is normally used as a primary screening tool to identify COVID-19 patients and was also the most common sign in both groups, consistently with other studies [13, 22]. All the other symptoms were equally reported among the two groups of patients, with the exception of dry cough that was more common in Group 2. Dry cough is typical of interstitial pneumonia and it may reflect the progressive severe pulmonary involvement of patients admitted to the ICU/SICU.

Overall, 63.2% of patients had pathological findings at the chest x-ray at admission, with no significant differences between the two groups. The homogeneous distribution of X-ray abnormalities may have been influenced by the exclusion of patients presenting a critical clinical condition from the beginning of the hospitalization. However, Group 2 patients were more likely to develop a pathological chest X ray during the hospital stay. These findings arouse two main observations. First the need of a close monitoring of patients in the early phase of the hospitalization and the suggestion that repeated radiological examinations are useful in identifying patients at risk for complications [23]. Second, the opportunity to consider a computed tomography (CT) chest scan as a first level diagnostic procedure in the context of COVID-19. To date, the best radiological strategy remains undefined. The use of CT-scan for all patients appears be unreasonable in terms of time, costs and radiation exposure [24]. An alternative option could be the combination of chest-X-ray and ultrasound, that has demonstrated a sensitivity of 75% (vs. 59% of chest X-ray) in detecting an interstitial lung disease [25, 26].

In terms of laboratory findings, Group 2 patients presented at admission a significantly higher WBC with neutrophilia, higher levels of AST and increased values of CRP. To identify possible early signals of clinical worsening and considering that the median time from hospital admission to the ICU/SICU transfer was of 5 days, we analysed the differences of laboratory findings at the 3rd measurement since admission: the differences between Group 1 and 2 identified at the beginning of the hospitalization were confirmed, with the inclusion of ALT and total bilirubin. However, WBC counts and hepatic inflammation markers resulted to be only moderately altered in Group 2. It is important to take into account that both high levels of CRP and the alterations of WBC and hepatic inflammation markers could be partly explained by concomitant bacterial infections and medications. Nevertheless, in our cohort, all the microbiological investigations aimed at the detection of bacterial superinfections resulted negative. Therefore it is reasonable to consider the persistent observation of even slightly abnormal WBC count and hepatic inflammation markers and of high levels of CRP as possible markers of disease severity that should lead physicians to a closer follow-up for the risk of clinical worsening. The association between high concentrations of CRP and COVID-19 severe clinical presentations has also been reported in several other studies [9, 10, 12, 16, 22].

A significant lower level of lymphocyte T CD4+ count was recorded in Group 2 compared to Group 1. Researchers have recently demonstrated that T cells can have a role in contrasting the SARS-CoV-2 infection and that the severity of the disease can depend on the strength of T cell responses [27, 28]. A speculation could be that the partial impaired immune system of Group 2 patients may play a detrimental role in the early phase of the infection, even thought it is still unknown whether T cells can promote the elimination of the virus or even cause a dangerous immune system overreaction [28].

In our cohort, patients transferred to the ICU/SICU resulted to be less likely to receive HCQ or CQ and Remdesivir during the course of hospitalization. On the other side, those patients were more often prescribed with Lopinavir/ritonavir, possibly because at the early stage of the outbreak this drug was the only one considered effective, and therefore more often used for severe patients. However, no conclusion can be drawn, and the effective role of these drugs used alone or in combination remains uncertain until the results of large controlled randomized studies will be available.

Our study as some limitations: first, the study cohort does not include COVID-19 patients admitted in general wards other than the Infectious Diseases Unit; a larger sample may have revealed additional elements not readily apparent in our series. Second, the observational design has the potential for incomplete capture or misclassification of baseline characteristics. Third, the follow up was not long enough to collect complete outcome data from all the patients.

However, the prospective collection of data, the monocentric design and the focus on the characteristics of the patients requiring either sub-intensive or intensive care represent added values.

## Conclusion

The proper management of COVID-19 patients requires a multilevel diagnostic approach that should be focused on the early recognition of signs of disease severity and progression. The clinical condition of patients primarily admitted to a general ward with a non-critical disease can worsen within few days, requiring the transfer to intensive or sub-intensive specialized units. The results of our study indicate that the presence of comorbidities, such as hypertension and diabetes, and of dry cough are potentially associated with a negative progression of the disease. In addition, not only laboratory alterations at admission, but also the persistent observation of abnormal laboratory findings should be regarded with a high index of suspicion for clinical worsening. The availability of tertiary level hospital with adequate provision of sub-intensive and intensive care is a crucial part of the response to the current and possible future outbreaks.

## Supplementary Information

**Additional file 1: Table 3.** Laboratory findings at 1°, 3° and last measurement of Group 1 and Group 2 patients.

## Data Availability

The Author confirms that the data supporting the findings of this study are available within the article. More information that supports the findings of this study is available from the corresponding author, AMC, upon reasonable request.

## Abbreviations

ALP: Alkaline Phosphatase
ALT: Alanine Aminotransferase
APTT: Activated Partial Thromboplastin Time
AST: Aspartate Aminotransferase
COVID-19: Coronavirus Diseases 2019
CQ: Chloroquine
CRP: C-Reactive-Protein
GFR: Glomerular Filtration Rate
GGT: Gamma-Glutamyl-Transferase
HCQ: Hydroxychloroquine
ICU: Intensive Care Unit
IDU: Infectious Diseases Unit
IQR: Inter-quartile Range
MV: Mechanical Ventilation
NIV: Non Invasive Ventilation
PT: Prothrombin Time
RT-PCR: Real Time Polymerase Chain Reaction
SARS-CoV-2: Severe Acute Respiratory Syndrome Coronavirus 2
SICU: Sub-Intensive Care Unit
