# Supplementary material for: Clinical characteristics and laboratory biomarkers changes in COVID-19 patients requiring or not intensive or sub-intensive care: a comparative study
Source: BMC Infect Dis. 2020 Dec 9;20:934. doi: 10.1186/s12879-020-05647-7 (PMC7724444; doi:10.1186/s12879-020-05647-7)
Supplement: Supplementary file 1 — Additional file 1: Table 3. Laboratory findings at 1°, 3° and last measurement of Group 1 and Group 2 patients. [file 12879_2020_5647_MOESM1_ESM.docx]

**Table 3.** Laboratory findings at 1°. 3° and last measurement of Group 1 and Group 2 patients.

|  | **1° measurement** | | | | **3° measurement** | | | | **Last measurement** | | | |
| --- | --- | --- | --- | --- | --- | --- | --- | --- | --- | --- | --- | --- |
| **Laboratory findings UOM (n.r.)** | **Avail** | **Group 1** | **Group 2** | **p** | **Avail** | **Group 1** | **Group 2** | **p** | **Avail** | **Group 1** | **Group 2** | **p** |
| Hb g/L (12.3-15.3) | 98.7% | 13.3  (12.3-14.4) | 12.9  (11.7-14.0) | 0.092 | 77.2% | 12.8  (11.6-13.9) | 12.3  (11.4-13.8) | 0.182 | 98.7% | 12.9  (11.7-14.1) | 9.7  (11.4-12.7) | <0.01 |
| WBC x10^9^/L (4.4-11) | 98.7% | 4.77  (3.69-6.8) | 6.25  (4.09-8.52) | 0.055 | 77.2% | 6.08  (4.19-7.93) | 8.16  (5.07-11.3) | <0.01 | 98.7% | 5.87  (4.55-7.44) | 7.85  (5.74-11) | <0.01 |
| Neutrophils x10^9^/L (1.8-7.8) | 98.7% | 3.24  (2.18-4.79) | 5.28  (2.77-7.28) | <0.01 | 71.6% | 3.96  (2.48-6.44) | 6.89  (4.44-9.35) | <0.01 | 98.7% | 3.38  (2.41-5.48) | 5.53  (3.44-7.96) | <0.01 |
| Lymphocytes x10^9^/L (1.1-4.8) | 98.7% | 102  (0.76-1.3) | 0.77  (0.52-1.09) | 0.242 | 71.6% | 1 (0.71-1.46) | 0.6  (0.46-0.97) | 0.084 | 98.7% | 1.28  (0.93-1.74) | 1.2  (0.82-1.81) | 0.86 |
| Platelet x10^9^/L (150-450) | 98.7% | 171  (135.5-216) | 167  (128-202) | 0.223 | 77.2% | 195  (142.2-268) | 210  (154-258) | 0.718 | 98.7% | 221 (141-291) | 237  (52-291) | 0.281 |
| AST U/L (10-35) | 98.7% | 32 (24-44) | 44 (33-65) | <0.01 | 71.6% | 33 (24-47) | 42  (29.5-71.5) | <0.01 | 98.7% | 28 (21-40.5) | 32 (24-40) | 0.589 |
| ALT U/L (7-35) | 98.7% | 26 (19-44) | 31 (20-56) | 0.181 | 71.6% | 31 (21-54) | 34  (19.5-82) | <0.01 | 98.7% | 32 (20.5-51) | 41 (27-68) | 0.115 |
| Bilirubin Total µmol/L (1.7-17) | 98% | 7.8  (5.7-11.5) | 9.2  (7.8-12.2) | 0.202 | 62.7% | 8.8 (8.6-11.2) | 12.1  (7.2-18.5) | <0.01 | 98% | 8.4 (6.1-10.9) | 9.6  (7.5-14.1) | 0.121 |
| GGT U/L (3-65) | 98% | 29 (17-55) | 38.5  (26-67.5) | 0.257 | 60% | 37 (20-71) | 44.5  (28.2-104) | 0.762 | 98% | 32 (19-59) | 56.5  (36.7-123) | 0.383 |
| ALP U/L (43-115) | 93.7% | 79.5  (59.5-104.7) | 62  (46-72.2) | 0.036 | 20.8% | 79.5  (59.5-104.7) | 62  (46-72.2) | 0.039 | 93.7% | 61 (49-79.5) | 68  (51-86.7) | 0.236 |
| CRP mg/dl (<6) | 92.1% | 43 (17-81.5) | 100  (45-140) | <0.01 | 62.4% | 34 (14-62) | 69 (30-130) | <0.01 | 73.9% | 15 (8.2-3.5) | 18  (7.9-67.5) | <0.01 |
| D-Dimer ug/l (<250) | 52.8% | 196.5  (158.7-268) | 217  (151-351) | 0.603 | 34.3% | 213  (168.2-328) | 373  (195.5-937) | 0.071 | 60% | 223  (168.5-310) | 277  (9.26-700) | 0.162 |
| Procalcitonin ug/l (<0.50) | 69.7% | 0.1 (0.1-0.2) | 0.21  (0.09-0.55) | 0.06 | 29.4% | 0.15  (0.09-0.37) | 0.21  (0.1-0.7) | 0.408 | 60% | 0.09 (0.05-0.2) | 0.14  (0.06-0.52) | 0.023 |
| BUN mmol/L (2.50-7.50) | 98% | 4.74  (3.9-6.6) | 6.1  (5.0-7.9) | 0.514 | 65.3% | 5.2 (3.6-8.5) | 7.4  (5.6-9.9) | 0.459 | 98% | 4.8 (3.6-7.1) | 6.9  (4.5-9.9) | <0.01 |
| Creatinin µmol/L (59-104) | 98.7% | 74 (64-89.5) | 86 (69-102) | 0.315 | 74.9% | 77 (68-92) | 80 (67-94) | 0.658 | 98.7% | 75 (64-91) | 75 (62-89) | 0.387 |
| GFR CPK-EPI ml/m’/1.73mq (90-120) | 72.6% | 92  (78.5-102) | 84 (67-94) | 0.03 | 46.5% | 90.5  (73.5-101) | 88  (69.5-98.5) | 0.472 | 74.9% | 93 (79-100.5) | 90 (73-100) | 0.176 |
| APTT s (22-32) | 95.7% | 24 (23-27) | 26 (24-30) | <0.01 | 51.5% | 23 (21-26.7) | 25 (21-27) | <0.01 | 94.4% | 23 (22-25) | 23 (20-26) | 0.682 |
| PT % (75-112) | 94.7% | 90.5 (81-99) | 86  (75.5-97) | 0.069 | 51.5% | 85 (75-96) | 82.5  (70-94.2) | 0.186 | 94.4% | 90 (80-99) | 85  (79.2-91.5) | 0.021 |
| CD4+ Lymph cell/ µL  (510-1270) | 34.6% | 469.5  (310-721) | 331.9  (140.2-575) | 0.031 | 1.6% | 352  (279.3-531.5) | 263.7 (165.7-361) | 0.463 | 34.5% | 517.4  (332.4-743.8) | 383.5 (156.7-575) | 0.052 |

Data presented as median (IQR). Data availability is also shown.

ICU: Intensive Care Unit; SICU: Sub-intensive Care Unit; IQR: Interquartile Range. Hb: Haemoglobin; WBC: White Blood Cells; AST: Aspartate Aminotransferase; ALT: Alanine Aminotransferase; GGT: Gamma Glutamyl Transferase; ALP: Alkaline Phosphatase; CRP: C Reactive Protein; BUN: Blood Urea Nitrogen; GFR CPK-EPI: Glomerular Filtration Rate Chronic Kidney Disease Epidemiology Collaboration; APTT: Activated Partial Thromboplastin Time; PT: Prothrombin Time.
